# Cost-effectiveness of replacing *versus* discarding the nail in children with nail bed injury

**DOI:** 10.1093/bjs/znad086

**Published:** 2023-04-18

**Authors:** Helen A Dakin, Thi Thu An Nguyen, Melina Dritsaki, Aina V H Greig, Jamie R Stokes, Jonathan A Cook, David J Beard, Loretta Davies, Matthew D Gardiner, Abhilash Jain, NINJA Collaborative, M E Png, A Jones, C Cooper, A Sierakowski, A Mertic, H Gerrish, K Cranmer, N Fox, P Dutta, G Vissers, P Costa, R Irri, G McArthur, M Horwitz, A Sleiwah, H Jephson, M Deeley, R Nicholas, Z Vinnicombe, A Nicola, C Bing Chuo, C Milner, J Heaney, J Totty, M Fleet, M Faheem Khadim, P Williams, S Bibawy, A Round, R Pinder, A Plonczak, G Lawton, D Kennedy, A Bennett, A Fadulelmola, J James, E Reay, K Beadon, T Cameron, Z Oliver, K Wensley, S Dupré, J Rodriguez, D Furniss, M Gale, A Knight, J Tulip, L Turner, L Wellings, M Allen, R Wade, V Itte, G Bourke, N Kumar, S O'Sullivan, J WM Jones, K Young, K Taylor, O Dawood, S Booth, L Giwa, R Pearl, A Coutts, R Hawkins, A Mostafa, T Nisbett, P Riddlestone, A Selby, C Uzoho, D Chasiouras, LC Bainbridge, T Buick, W Lam, B Baker, K Walsh, K Keating, R Dalan, M Shah, D Mead, S Diment, M Nicolau, B Smeeton, D Thomson, N Senior, J Moledina, J Colville, K Manso, M Song, O Manley, P Drury, R Kerstein, W Cobb, J Wormald, R Shirley, A Tan, A Arnaout, C Cruz, N Brice, N Segaren, N Joji, R Chawla, S Hassanin, R Adami, H Ridha, A Cook, L Symington, R Long, S Dustagheer, H Jarvis, M Larsen, M Williams, R Trickett, D Miles, A Pai, C Honeywell, C Brady, S Madhavan, V Manou, G Phillips, R Baker

**Affiliations:** Health Economics Research Centre, Nuffield Department of Population Health, University of Oxford, Oxford, UK; Department of Economics and Related Studies, University of York, York, UK; Nuffield Department of Orthopaedics, Rheumatology and Musculoskeletal Sciences, University of Oxford, Oxford, UK; Department of Economics and Laboratory of Applied Economics, University of Western Macedonia, Kastoria, Greece; Department of Plastic and Reconstructive Surgery, Guy’s and St Thomas’ NHS Foundation Trust, London, UK; Nuffield Department of Orthopaedics, Rheumatology and Musculoskeletal Sciences, University of Oxford, Oxford, UK; Nuffield Department of Orthopaedics, Rheumatology and Musculoskeletal Sciences, University of Oxford, Oxford, UK; Nuffield Department of Orthopaedics, Rheumatology and Musculoskeletal Sciences, University of Oxford, Oxford, UK; Nuffield Department of Orthopaedics, Rheumatology and Musculoskeletal Sciences, University of Oxford, Oxford, UK; Nuffield Department of Orthopaedics, Rheumatology and Musculoskeletal Sciences, University of Oxford, Oxford, UK; Department of Plastic Surgery, Wexham Park Hospital, Frimley Health NHS Foundation Trust, Slough, UK; Nuffield Department of Orthopaedics, Rheumatology and Musculoskeletal Sciences, University of Oxford, Oxford, UK; Department of Plastic Surgery, Imperial College NHS Healthcare Trust London, London, UK

## Introduction

Approximately 10 000 operations to repair paediatric nail bed injuries are conducted in the UK each year (based on a multicentre service evaluation^1,2^). The optimal management of nail bed injuries has been debated^3–6^. Surgical treatment involves removing the nail plate (fingernail) and using sutures to repair the underlying nail bed laceration. Following the repair, 96 per cent of UK surgeons currently replace the nail plate under the proximal nail fold^1^, aiming to protect the nail bed repair site.

The UK-based NINJA (Nail bed INJury Analysis) trial (ISRCTN44551796; National Research Ethics Committee 18/SC/0024) randomized 451 children aged under 16 years to either replacing or discarding the nail plate after nail bed injury repair^2,7^. It was found that the nail replacement group had non-significantly more infections than the discard group and similar cosmetic appearance of the nail^2^. No studies have previously assessed the cost-effectiveness of discarding or replacing the nail after nail bed injuries in children.

This report presents the results of a within-trial economic evaluation conducted as part of the NINJA trial, which assessed cost-effectiveness of nail replacement *versus* nail discard after nail bed repair in children.

## Methods

The primary analysis comprised a cost-effectiveness analysis estimating the cost per infection avoided^8^. The base-case analysis included infections within 7–10 days of nail bed repair, because this comprised the primary trial endpoint and was collected for almost all patients during a routine clinic visit. However, as nail injuries can take up to 4 months to recover fully^9^, the base-case analysis included costs related to the nail injury that arose before the final follow-up, which took place 4–12 months after nail bed repair.

A cost-utility analysis with a 4-month time horizon was also conducted, which estimated the cost per quality-adjusted life-year (QALY) gained; this comprised a secondary analysis because no utility instruments are available for children under 2 years of age and there is no validated tariff for the EQ-5D-Y™ questionnaire (EuroQol Group, Rotterdam, the Netherlands)^10^.

The base-case analysis took a UK National Health Service (NHS) perspective, focusing on direct healthcare costs; a sensitivity analysis took a broader societal perspective. Resource use was measured using trial case report forms and parent-completed questionnaires, and was costed in 2019 UK pounds using NHS tariffs^11–17^. The methods are described in more detail in the *Supplementary Methods*, including unit costs (*Table S1*) and how missing data were handled using multiple imputation (*Table S2*).

## Results

Nail replacement required an additional suture (assumed to cost £4.27) and extended operating time by a mean of 3.24 (95 per cent c.i. 1.34 to 5.13) min (*P* < 0.001) (*Supplementary Results* and *Table S3*). Of the patients with complete follow-up data, 6 of 105 (5.7 per cent) in the replace group had some NHS consultations other than the 7-day check, compared with 15 of 99 (15 per cent) in the discard group.

The mean cost of prescribed painkillers and consultations with healthcare professionals was £7.34 (95 per cent c.i. –10.70 to 25.55) higher in the replaced group (*P* = 0.422) (*Table 1*). The mean cost of antibiotics (*P* = 0.773) and 7-day check-up (*P* = 0.757) did not differ significantly between groups. One patient in the replace group had a severe infection requiring further surgery, considered probably related to the treatment allocation^2^, which led to treatment costs of £2542; the mean cost of managing serious adverse events was therefore £11.20 (0 to 33.60) higher in the replace group (*P* = 0.773). There was no significant difference between groups in non-NHS costs, such as lost income, over-the-counter medications, travel, and childcare (*P* = 0.720). The mean total NHS cost during the trial period was £75.07 (30.05 to 124.11) per patient lower in the discard group than the replace group (*P* < 0.001).

**Table 1:** Results of economic evaluation during trial period up to final follow-up 4–12 months after nail bed repair surgery, based on bootstrapping and multiple imputation

	Nail replaced (*n* = 227)	Nail discarded (*n* = 224)	Difference (replace minus discard)
Cost of suture for replacing nail (£)*	3.94 (3.78, 4.07)‡	0.08 (0.02, 0.15)‡	3.86 (3.69, 4.01)‡
Cost of sutures for nail bed repair (£)	5.26 (4.90, 5.64)‡	5.08 (4.75, 5.44)‡	0.18 (−0.32, 0.69)
Cost of operating time (£)	359.65 (337.32, 382.98)‡	304.02 (284.11, 324.01)‡	55.62 (25.34, 85.67)‡
Cost of 7-day check (£)†	195.16 (188.94, 200.53)‡	194.11 (187.76, 199.56)‡	1.05 (−7.16, 9.15)
Cost of antibiotics (£)	3.60 (2.99, 4.25)‡	3.74 (3.05, 4.53)‡	−0.14 (−1.14, 0.79)
Cost of managing related serious adverse events (£)	11.20 (0, 33.60)	0 (0, 0)	11.20 (0, 33.60)
Cost of NHS consultations and prescribed painkillers (£)	23.53 (11.64, 38.17)‡	16.19 (6.53, 29.69)‡	7.34 (−10.70, 25.55)
Non-NHS cost (£)	130.28 (85.38, 184.21)‡	142.14 (97.38, 198.77)‡	−11.86 (−82.62, 54.14)
Total cost from NHS perspective (£)	593.14 (558.21, 635.69)‡	518.07 (493.73, 543.97)‡	75.07 (30.05, 124.11)‡
Total cost from societal perspective (£)	723.42 (664.42, 790.85)‡	660.22 (606.74, 723.89)‡	63.21 (−21.81, 147.12)
Rate of infections by 7–10 days per patient	0.0230 (0.0044, 0.0441)‡	0.0093 (0, 0.0223)	0.0137 (−0.0091, 0.0352)
QALYs gained by 4 months among patients aged ≥ 2 years (158 nail replaced; 165 nail discarded)	0.2826 (0.2733, 0.2907)‡	0.2859 (0.2777, 0.2934)‡	−0.0034 (−0.0150, 0.0082)

Values are mean (95% c.i.). *Each suture cost £4.27 and was applied to all patients who had the nail replaced, regardless of treatment allocation. Owing to deviations from the randomized allocation, the cost in the replace group was therefore slightly lower than £4.27 and the cost in the discard group was above £0. †This normally comprised a plastics outpatient consultation. A minority of patients did not attend this consultation (in which case no cost was applied); for even fewer patients, the 7-day check was conducted by a practice nurse or general practitioner as indicated in the notes. QALY, quality-adjusted life-year. ‡*P* < 0.050 based on non-parametric bootstrapping.

After imputing missing data, 2.3 (95 per cent c.i. 0.4 to 4.4) per cent of patients in the replace group and 0.9 (0 to 2.2) per cent of those in the discard group had nail bed infections within 7–10 days of surgery, a difference of 0.0137 (−0.0091 to 0.0352) infections per patient treated (*P* = 0.338).

**Fig. 1:**
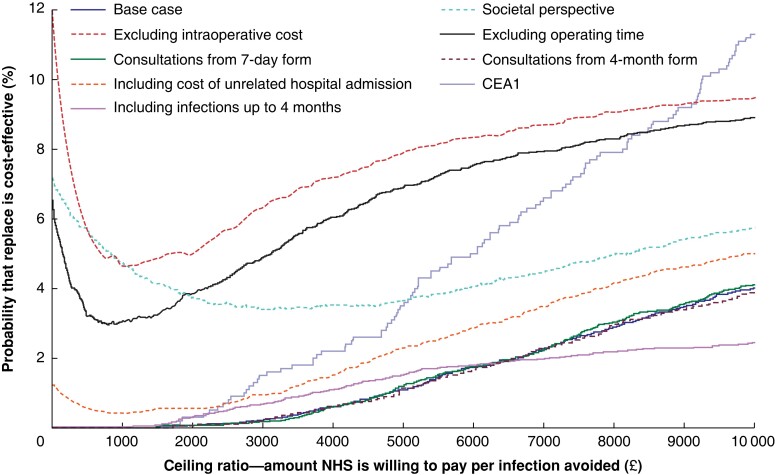
Cost-effectiveness acceptability curves for base-case and sensitivity analyses This shows the probability that replacing the nail is good value for money at different estimates of the amount that the National Health Service (NHS) might be willing to pay to avoid one nail bed infection. It quantifies the uncertainty there is around the conclusions, given the trial results and the lack of evidence about how much it is worth paying to avoid one infection. To highlight differences between analyses, the *y*-axis shows only the 0–12% range. Cost-effectiveness analysis 1 (CEA1) comprised a complete-case analysis (excluding any patients with missing data on 7-day infections or resources at 7–10 days), took a 7–10-day time horizon, and excluded the cost of all drugs (including antibiotics).

Discarding the nail during nail bed repair surgery is therefore dominant over nail replacement, having significantly lower costs and numerically fewer infections. In bootstrapping analyses, the probability that replacing the nail is both more costly and less effective is 82.91 per cent, and there is a 99.95 per cent probability that discarding the nail will save money (*Figs 1* and *S1*). The probability that replacing the nail is cost-effective is below 4 per cent at any ceiling ratio between £0 and £10 000 per nail infection avoided; one can therefore be over 96 per cent confident that discarding the nail represents better value for money than replacing it (*Fig. 1*). Discarding the nail represents statistically significantly better value for money unless the NHS is willing to pay more than £7300 per nail bed infection avoided (*P* < 0.050). Sensitivity analyses confirmed the base-case conclusions (*Fig. 1* and *Table S4*).

For patients aged at least 2 years, mean EQ-5D-Y™ utility was 0.70 immediately before the nail bed repair operation, and rose to 0.76 by day 7 and 0.96 by 4 months after surgery (*Table S5*). There was no significant difference in EQ-5D-Y™ utility at any time point (*P* ≥ 0.489). The replace group accrued a mean of −0.0034 (95 per cent c.i. −0.0150 to 0.0082) fewer QALYs than the discard group (*P* = 0.670) (*Table 1*). Bootstrapping showed that there is a 12 per cent probability of replace being cost-effective if the NHS is willing to pay £20 000 per QALY gained (*Table S6*, *Figs S2* and *S3*).

## Discussion

Both the cost-effectiveness and cost–utility analyses show that discarding the nail when treating nail bed injury in children is less costly and at least as effective as the current practice of replacing the nail. However, there is uncertainty around this conclusion as there was no significant difference in health outcomes. Although costs to patients’ families were numerically higher in the replace group, the variability in non-NHS costs meant that there was no significant difference in costs from a societal perspective. The probability that nail replacement is cost-effective depends on how much the NHS is willing to pay to avoid one nail bed infection, which is not known; if society is willing to pay no more than £10 000 (€11 000, https://www.xe.com 31 March 2023) per infection avoided, there is an at least 96 per cent probability that discarding the nail is cost-effective. Nonetheless, the NINJA trial demonstrates that discarding the nail is significantly cheaper than nail replacement, and the 95 per cent confidence interval around the incidence of infections excludes a 1 per cent difference in favour of nail replacement.

Although several areas of uncertainty remain, the current practice of nail replacement could be discontinued if the decision could not be deferred while more evidence is collected. With the knowledge available at present, budget allocation decisions should rely on the mean net benefits, and adopt technologies that are beneficial on average even though they are not statistically significant^18^.

As healthcare costs were £75 (€85) per patient lower with discarding the nail, the UK NHS could save £720 000 (€819 000) per year by discarding the injured nail for the 9600 children who currently have the nail replaced after nail bed repair each year^1^.

To the authors’ knowledge, this is the first trial-based economic evaluation to compare the cost-effectiveness of approaches to treating nail bed injuries in children. The analysis demonstrates that discarding the nail is cheaper and does not reduce quality of life. Given these results, there is economic justification for recommending that the nail should be discarded rather than replaced after repair of nail bed injury in children.

## Collaborators

NINJA Collaborative: M. E. Png, A. Jones, C. Cooper (University of Oxford, UK); A. Sierakowski (Mid and South Essex NHS Foundation Trust, Chelmsford, UK); A. Mertic, H. Gerrish, K. Cranmer, N. Fox, P. Dutta (Broomfield Hospital, Chelmsford, UK); G. Vissers, P. Costa, R. Irri, G. McArthur, M. Horwitz (Chelsea and Westminster Hospital, London, UK); A. Sleiwah, H. Jephson, M. Deeley, R. Nicholas, Z. Vinnicombe, A. Nicola (Guys and St Thomas' Hospitals, London, UK); C. Bing Chuo, C. Milner, J. Heaney, J. Totty, M. Fleet, M. Faheem Khadim, P. Williams, S. Bibawy, A. Round, R. Pinder (Hull Royal Infirmary, Hull, UK); A. Plonczak, G. Lawton, D. Kennedy (St Mary's Hospital, London, UK); A. Bennett, A. Fadulelmola, J. James, E. Reay (James Cook Hospital, Middleborough, UK); K. Beadon, T. Cameron, Z. Oliver, K. Wensley, S. Dupré, J. Rodriguez, D. Furniss (John Racliffe Hospital, Oxford, UK); M. Gale (Kings Mill Hospital, Sutton in Ashfield, UK); A. Knight, J. Tulip, L. Turner, L. Wellings, M. Allen, R. Wade, V. Itte, G. Bourke (Leeds General Infirmary, Leeds, UK); N. Kumar, S. O'Sullivan, J. WM Jones (Peterborough City Hospital, Peterborough, UK); K. Young, K. Taylor, O. Dawood, S. Booth, L. Giwa, R. Pearl (Queen Victoria Hospital, East Grinstead, UK); A. Coutts, R. Hawkins, A. Mostafa, T. Nisbett, P. Riddlestone (Royal Cornwall Hospital, Truro, UK); A. Selby, C. Uzoho, D. Chasiouras, LC. Bainbridge (Royal Derby Hospital, Derby, UK); T. Buick, W. Lam (Royal Hopsital for Sick Children, Edinburgh, UK); B. Baker, K. Walsh, K. Keating, R. Dalan, M. Shah (Royal Manchester Children's Hospital, Manchester, UK); D. Mead, S. Diment, M. Nicolau (Salisbury District Hospital, Salisbury, UK); B. Smeeton, D. Thomson, N. Senior, J. Moledina, J. Colville (St George's Hospital, London, UK); K. Manso, M. Song, O. Manley, P. Drury, R. Kerstein, W. Cobb, J. Wormald, R. Shirley (Stoke Mandeville Hospital, Aylesbury, UK); A. Tan, A. Arnaout, C. Cruz, N. Brice, N. Segaren, N. Joji, R. Chawla, S. Hassanin, R. Adami, H. Ridha (The Lister Hospital, Stevenage, UK); A. Cook, L. Symington, R. Long, S. Dustagheer (The Ulster Hospital, Belfast, UK); H. Jarvis, M. Larsen, M. Williams, R. Trickett (University Hospital of Wales, Cardiff, UK); D. Miles (University of Essex, Colchester, UK); A. Pai, C. Honeywell, C. Brady, S. Madhavan, V. Manou, G. Phillips, R. Baker (Wexham Park Hospital, Slough, UK).

## Supplementary Material

znad086_Supplementary_Data

## Data Availability

Requests to access the data set from qualified researchers trained in human subject confidentiality protocols may be sent to SITU at situ@ndorms.ox.ac.uk. For the purpose of open access, the authors have applied a Creative Commons Attribution (CC-BY) licence to any Author Accepted Manuscript version arising from this submission.
