# Supplementary material for: Cost-effectiveness of replacing versus discarding the nail in children with nail bed injury
Source: Br J Surg. 2023 Apr 18;110(9):1104–7. doi: 10.1093/bjs/znad086 (PMC10416701; doi:10.1093/bjs/znad086)
Supplement: znad086_Supplementary_Data [file znad086_supplementary_data.zip › Supplementary_Material.docx]

**Cost-effectiveness of Nail Replacement Compared with Discarding the Nail in Children with Nail Bed Injury**

Helen A. Dakin,^1*^ Thi Thu An Nguyen,^2*^ Melina Dritsaki,^3,4^ Aina V.H. Greig,^5^ Jamie R. Stokes,^3^ Jonathan A. Cook,^3^ David J Beard,^3^ Loretta Davies,^3^ Matthew D. Gardiner,^3,6^ Abhilash Jain,^3,7^ and the NINJA Collaborative

* These authors contributed equally

1. Health Economics Research Centre, Nuffield Department of Population Health, University of Oxford, Oxford, UK

2. Department of Economics and Related Studies, University of York, York, UK

3. Nuffield Department of Orthopaedics, Rheumatology and Musculoskeletal Sciences, University of Oxford, Oxford, UK

4. Department of Economics & Laboratory of Applied Economics, University of Western Macedonia, Kastoria, Greece.

5. Department of Plastic and Reconstructive Surgery, Guy’s and St Thomas’ NHS Foundation Trust, London, UK

6. Department of Plastic Surgery, Wexham Park Hospital, Frimley Health NHS Foundation Trust, Slough, UK

7. Department of Plastic Surgery, Imperial College NHS Healthcare Trust London, London, UK.

**Contact details for corresponding author:**

**Helen Dakin,** Health Economics Research Centre, Nuffield Department of Population Health, University of Oxford, Old Road Campus, Headington, Oxford, OX3 7LF, UK. Email: helen.dakin@dph.ox.ac.uk; Tel: 01865 289422. <http://orcid.org/0000-0003-3255-748X>

**Supplementary Materials - Index**

| **Supplementary Methods** | *pag. 3* |
| --- | --- |
| Overview | *pag. 3* |
| Measuring costs | *pag. 3* |
| List of assumptions used within the analysis | *pag. 4* |
| Cost-utility analysis | *pag 6* |
| Missing data and uncertainty | *pag 7* |
| Sensitivity analyses | *pag. 10* |
| **Supplementary Results** | *pag. 11* |
| Results of sensitivity analyses on the cost-effectiveness analysis | *pag. 11* |
| Results of the cost-utility analysis | *pag. 11* |
| Strengths and weaknesses of the study | *pag. 11* |
| **Supplementary Figures and Tables** |  |
| **Table S1:** Unit costs | *pag. 13* |
| **Table S2:** Multiple imputation model specification | *pag. 15* |
| **Table S3:** Costs and resource use calculated on an available case basis | *pag. 16* |
| **Figure S1:** Scatter graph showing the base case economic evaluation results on the cost-effectiveness plane | *pag. 18* |
| **Table S4:** Results of the sensitivity analyses on the cost-effectiveness analysis | *pag. 19* |
| **Table S5:** Result for EQ-5D-Y | *pag. 20* |
| **Table S6:** Results of the cost-utility analysis and sensitivity analyses | *pag. 21* |
| **Figure S1:** Scatter graph showing the results of the cost-utility analysis on the cost-effectiveness plane | *pag. 22* |
| **Figure S2:** Cost-effectiveness acceptability curves for the cost-utility analysis | *pag. 23* |
| **References** | *pag. 24* |

**Supplementary methods**

***Overview***

The economic evaluation was pre-specified in a published statistical and health economic analysis plan submitted for publication before analysis began.^1^ The analysis followed the analysis plan, except that the cost of operation time was included in the analysis and three additional sensitivity analyses were conducted in addition to those specified in the plan.

The interventions compared in the analysis were chosen to match those compared in the NINJA trial. The economic evaluation focused on a UK setting, where a decision must be made about whether or not to replace the nail following repair of a nail bed injury.

NINJA and its pilot^2^ are to date the only randomised trials evaluating nail bed injuries in children and are therefore the only available source of robust clinical effectiveness data.

Since the time horizon was less than one year, discounting of cost and outcomes was unnecessary.

## ***Measuring costs***

Resource use related to the nail bed injury was measured using trial case report forms completed at the time of the operation, and after 7-10 days, and from questionnaires completed by parents at approximately 7 days and at the final follow-up (4-12 months post-surgery). Direct healthcare costs included the nail bed repair operation, seven-day check-up/dressing change, prescribed medication, healthcare consultations and managing serious adverse events related to the intervention. The secondary analysis taking a societal perspective also included indirect costs: namely the cost of days off work, over-the-counter medication, travel and childcare.

The cost of the nail bed repair operation was based on the cost of sutures and operation time (which was assumed to include staff, overheads, anaesthetic, antiseptic, dressings and intraoperative antibiotics^3^). We assumed intra-operative costs were the same for both groups, except for time in theatre and one extra suture used to reattach the nail in patients who had nail replacement.

Medications and healthcare consultations related to the nail bed injury were reported by parents. We also included the cost of managing serious adverse events (SAEs) that were considered by investigators to be related to the study intervention. Only one related SAE was reported in NINJA^4^; the detailed cost of this event was valued manually to avoid any duplication between the questionnaire and the SAE report.

Unit costs were obtained from NHS tariffs,^3,5-8^ supplemented by other sources where necessary^9,10^ (Table S1). Costs are presented in 2019 UK pounds (£).

***List of assumptions used within the analysis***

*Operation*

- We assumed that the cost of operation time^3^ captured staff, overheads, anaesthetic, antiseptic, dressings and intraoperative antibiotics.
- Suture costs were included on top of the cost of operation time to make sure that the analysis captured the cost of the additional suture that is required for nail bed repair as well as the cost of staff and overheads. We assumed that the cost of sutures would account for only a small proportion of the cost of operation time and therefore that double counting would be minimal.
- Sutures used for the nail bed repair itself were costed based on reverse cutting needle and assumed a 45cm length (unless operative forms indicated otherwise). When the suture type was not specified, we assumed to be the most commonly used Vicryl Rapide suture in the dataset (across both study groups).
- We assumed that the cost of dressings were included in the cost of the seven-day check and any other consultations in which dressing changes were conducted.

*7-day check-up*

- The cost of the 7 day clinic visit was included in the analysis and costed as a ‘outpatient visit at a Plastic or hospital dressing clinic’ whenever the 7-10 day clinic visit form was completed, except when free text fields stated that the patient had a 7-day check at the GP surgery or states that they did not attend 7-day visit. No cost was applied for this consultation for patients when the seven-day form was missing, or when the notes state that they did not attend 7-day visit.

*7-day/4-month forms*

- The 7-day health resource form could be completed either before or after the date of the 7-day check-up/dressing change. Whenever the 7-day health resource form was dated on or after the date of the 7-day check, we reduced the number of plastics visits reported on the health resource form by one to avoid double-counting, based on the assumption that the plastics visits that was reported on the resource form is the 7-day check-up rather than a separate visit. No adjustment was made if no plastics visits were reported on the 7-day health resource form.
- If a parent ticked yes for outpatient visits, but didn’t tick anything else, we applied cost of 1 plastics visit.
- If a parent ticked yes for outpatient visits, and ticked some but not all subcategories, we assumed the patient did not use any of the subcategories that were not ticked.
- If the parent said they had some community health care resource use but didn’t say what, we assumed it was a practice nurse consultation
- if parents ticked “NHS” for plastics/emergency/GP/physio/nurse provider and didn't fill in number of visits, assumed 1 NHS plastics consultation
- 2 phone calls to the hospital or dressing/children’s department and one case where medications were recorded as “call” were costed as 111 calls
- For one patient who had complete information on outpatient visits but not the page on community visits, we used the conditional mean imputation for the cost of community consultations, taking average across patients in that treatment group. This avoided the need to over-complicate multiple imputation.

*Medication*

- Data on antibiotic prescriptions in the 1^st^ 7 days were available from 3 different forms. We use the information from the 3 forms to identify the antibiotics that each patient received. For instance, if a drug is mentioned in both clinic form and 7-day health care resource form, it was listed as one usage as patients usually have one course of medication or switch to other alternatives.
- For antibiotics, we assumed that if patients were prescribed 2 or more different post-operative antibiotics, NHS would accrue the cost of both prescriptions. Therefore, the cost of antibiotics for any patients would be the sum of all antibiotics that have been prescribed. In most cases, having 2 different antibiotics listed on 2 different forms would suggest that the patient switched antibiotic. However, the operative form asks for the name of the *intraoperative* antibiotic and the duration of the *post-operative* antibiotics, but doesn't specifically ask for the name of the post-operative antibiotic. If the operative form says the intraoperative antibiotic was X1 and the 7 day clinic form says that the post-operative antibiotic was X2 (and the 7 day and 4 month questionnaires were either missing or also said X2), we would assume that X2 was the only post-operative antibiotic prescribed. However, if the 7 day clinic form said X1 and the 7 day patient form said X2, we would assume that they had both X1 and X2.
- If one or more of the case report forms stated that the patient received antibiotic X1, whereas one or more other case report forms did not mention any antibiotic prescriptions, we applied the cost of antibiotic X1. This may arise because a patient wasn't prescribed prophylactic antibiotics, but started a course a day or 2 after the operation. It may also arise because the patient was prescribed prophylactic antibiotics straight after the operation, but the parents forgot to put it on the seven-day form or didn't return the seven-day form.
- If the same antibiotic was mentioned on more than one form, we applied the cost of 1 prescription for that antibiotic, unless it was indicated on case report forms that the patient did receive 2 or more courses of X1.
- In general, we assumed that the post-operative antibiotic was the same as the intraoperative antibiotic recorded on the operation form.   However, if pts got IV cefuroxime during operation, we assumed that they would have a bottle of co-amoxiclav to take orally at home unless the other case report forms suggest that a different oral antibiotic was given.
- Medications were costed as one bottle of oral suspension for all age groups to simplify the analysis. Although it is likely that adolescents may receive tablets and smaller children may receive a lower concentration solution, while larger children may require 2 bottles of medication, antibiotics prescriptions account for only a small proportion of total costs and were similar in the 2 groups, so this simplification is unlikely to change the conclusions.
- For any use of paracetamol or ibuprofen, we applied the cost of a 100ml bottle of the dose appropriate for the child’s age (e.g. <6 or 6+) for each child. This is a reasonable reflection of how much it would actually cost the NHS to prescribe paracetamol/ibuprofen and is an approximation of the potential cost to parents.

## ***Cost-utility analysis***

NINJA initially included two health-related quality of life instruments: EQ-5D-Y^11^ and Paediatrics Quality of Life Inventory (PedsQL).^12,13^ However, the protocol was amended to remove PedsQL partway through the trial, because there is no mapping valid for children under seven years,^14^ making EQ-5D-Y the sole instrument used. EQ-5D-Y was self-completed by children aged seven years and older, while parents completed EQ-5D-Y (proxy) for children aged 2-6 years; there was no utility measurement for children under two years as there are no validated preference-based health-related quality-of-life (HRQoL) measures for this age group.^15^ Since there is no validated tariff for estimating EQ-5D utility based on the EQ-5D-Y, we used the UK time trade-off tariff for the adult version of the EQ-5D questionnaire.^16^

We estimated QALYs for each patient aged two years and older from EQ-5D utilities as the area under the curve for the three utility (HRQoL) measurements, assuming that utility changes in a straight line between each measurement:

$$QALYs =\left( \frac{{HRQoL}_{baseline}+ {HRQoL}_{7d}}{2} \right)*\frac{7}{365} +\left( \frac{{HRQoL}_{7d}+ {HRQoL}_{4m}}{2} \right)*\left( \frac{4}{12}- \frac{7}{365} \right)$$

The 7-10 day questionnaires were assumed to be completed exactly 7 days after the operation and the 4-12 month questionnaires were assumed to be completed exactly 4 months after to avoid the risk of bias. To adjust for chance imbalance in baseline utility, the mean QALYs between groups were estimated by regressing QALYs on treatment allocation and baseline utility.^17^

## ***Missing data and uncertainty***

We handled missing data with multiple imputation, in which missing values were replaced with more than one predicted value regressed on available observations.^18^ Imputation was conducted separately for the cost-effectiveness analysis and the cost-utility analysis as cost-utility analysis excluded children under two. For the cost-effectiveness analysis, 55 sets of imputed values were estimated, since 55% of patients had missing data in at least one variable; 65 imputed datasets were estimated for the cost-utility analysis.

Each imputed dataset was bootstrapped 100 times and the 5500-6500 sets of results were pooled together, which is equivalent to Rubin’s rule.^19,20^ For each bootstrap on each imputed dataset, we estimated the mean cost in each group (both for total cost and key cost components) as well as either the proportion of patients with infections, or mean QALYs (adjusted for baseline EQ-5D utility). We calculated 95% confidence intervals as the 2.5^th^ and 97.5^th^ percentiles across the 5500-6500 bootstraps and plotted the results on a scatter graph.

The chance that replacing the nail is cost-effective depends on how much the NHS is willing to pay for an additional unit of health gain. When interpreting cost-utility analysis results, we used a £20,000/QALY ceiling ratio, since the National Institute for Health and Care Excellence (NICE) consider treatments costing less than £20,000-£30,000/QALY to be cost-effective. For the cost-effectiveness analysis, we present the probability that nail replacement would be cost-effective at a range of arbitrary ceiling ratios ranging between £0 and £10,000 per infection avoided. This range was assumed to include the maximum value that the NHS might be willing to pay to avoid a localised infection of this type. We plotted cost-effectiveness acceptability curves representing the probability that replacing the nail is cost-effective at different levels of the threshold.

Chained regression equations were used as pre-specified^1^ and recommended for non-normal variables.^21^ The other approach, multivariate normal imputations (MI-JM), is not less appropriated since it requires imputation model to be jointly normally distributed, which is not plausible as both cost and health outcomes are generally non-normally distributed.^22^ The validity of model depends on its specification, referring the inclusion and exclusion of conditional variables.^23^

The validity of multiple imputation depends on the correct specification of the imputation model, which is often referred to as the inclusion and exclusion of conditional variables.^23^ In general, the model should include all variables involving in the missing mechanism and the complete data analysis.^21^ However, including too many variables, such as cost components in the same questionnaire, would probably lead to the problem of perfect prediction as these variables are likely to share the same missing pattern.^18^

Our imputation model imputed aggregated cost and health outcomes based on baseline covariates and treatment allocation to avoid perfect prediction and an over-complicated model (Table S2). Each variable was predicted by all others listed variables in an iterated process of regression.

We imputed consultation costs from both the 7-day and 4-month questionnaires, which asked parents to complete resource use since the operation. However, because few patients accrued costs beyond day 7, recall bias meant that the costs in these questionnaires were similar. The consultation and medication costs for each patient were therefore based on the questionnaire with the highest cost for that patient, in order to include resource use throughout the time horizon.

For the cost-effectiveness analysis, the imputation model was the iterated process of predicting cost and 7-day infection based on each other and independent variables (Table S2). Except where stated, all regressions were simple linear regression with predictive mean matching to choose the closest to the predicted value from the real sample. As infection is a binary outcome, logistic regression was applied. However, categorical outcomes combined with a predictor with mainly zero value can result in perfect prediction.^21^ We encountered this problem in this trial as infection rate 7-day and 4-month were low and no patients in the discard group developed new infections between 7 days and 4 months. Thus, 4-month infection was omitted completely from the model to avoid perfect prediction and ensure that we could keep treatment allocation as a predictor. Other variables that caused problems with the perfect prediction of 7-day infection were omitted in specific regression. For instance, NHS cost as a perfect predictor of infection was omitted in the regression of infection but still predicted other missing costs.

The same iterated process was repeated for cost-utility analysis, but patients aged under 2 years were excluded before imputation. In line with recommendations,^21^ missing values for baseline EQ-5D utilities were imputed deterministically as the overall mean baseline utility (across both groups) to make sure that imputation did not introduce any imbalance between groups in pre-randomisation variables. Infections were excluded from the imputation function due to perfect prediction; all regressions were therefore linear with predictive mean matching.

### *Imputation process:*

1. Imputation step: M sets of imputed values are generated, creating M completed data set. The appropriate number of imputations depends on missing variables and the data of each trial. Despite there has not been any formal recommendation to choose, the number of imputations equals the percentage of patients with any missing data was the general rule of thumb.^21^ Therefore, we applied 55 imputations for CEA2 and 65 imputations for CUA based on the proportion of patients with missing data on any variable.
2. Complete data analysis step: Multiple imputation generated M complete sets of data on the outcomes and cost components shown in Table S2. The recall periods in the 7-day and 4-month questionnaires overlapped completely as both ask for resource use since the operation. Exploratory data analysis showed that the cost of the total from the 4-month resource use form was lower than that from the 7-day resource use for 33.8% (68/201) of patients who completed both questionnaires and was the same for 57.7% (116/201). This may be because the 4-month form is subject to recall bias where patients might list service use less accurately from the previous form.^24,25^. Therefore, we assumed that the used whichever post-imputation data include higher values of NHS and non-NHS cost of participants either had or missing both questionaries. Therefore, we utilised real data whenever possible and used whichever value was higher if patients either returned both questionnaires or were missing both questionnaires. For example, if observations had either 7-day and 4-month data, this data was used instead of imputed value. The same mechanism applied to the case when both forms were available. Then total cost in both NHS and societal perspective were added as the sum of relevant cost components, including 3 cost components with complete data that had been omitted from multiple imputations: cost of treating serious adverse events; 7-day clinic cost; and cost of sutures for reattaching the nail.
3. Pooling step: Each imputation was independently bootstrapped 100 times to create 5500 and 6500 complete data sets for CEA2 and CUA respectively. There are several methods to pool bootstraps and combine them into one result. Rubin’s rule is one of the most common approaches, which sums up the imputation uncertainty variation and the uncertainty around the coefficient of the estimated parameter.^26^ However, recent research comparing Rubin’s rule and the direct standard deviation across bootstraps suggest that the standard error and confident interval of the target parameter is virtually identical when more than 20 imputations are being pooled.^19,20^ Since we had >50 imputations, the pooled sample approach should give identical results to Rubin’s rule and is simpler to apply and facilitates the presentation of decision uncertainty. Therefore, we adopted the latter approach, which was also recommended by Schomaker.^19^

***Sensitivity analyses***

We conducted the following sensitivity analyses to assess the effect of changing the assumptions or methods on cost-effectiveness:

- Societal perspective
- Excluding intra-operative cost: includes the cost of all of the resources from the 7 day form and the cost of the 7 day check, but excludes sutures and operation time
- Excluding operation time: Includes all costs that are in the base case analysis except operation time (matching statistical and health economics analysis plan.^1^
- Using consultation and drug data solely from 7-day form
- Using consultation and drug data solely from 4-month form
- Including cost of unrelated hospital admission: This is identical to the base case analysis, except for including the cost of one paediatric hospital admission for 1 patient in the discard group who was hospitalised for a week for an SAE related to the operation, but not related to the intervention.
- CEA1: cost-effectiveness analysis 1 (CEA1) specified in the statistical and health economics analysis plan^1^ comprised a complete case analysis (excluding any patients with missing data on 7-day infections or resources at 7-10 days), took a 7-10 day time horizon and excluded the cost of all drugs (including antibiotics).

**Supplementary results**

***Results of sensitivity analyses on the cost-effectiveness analysis***

Sensitivity analyses confirmed the base case conclusions (Table S4). Taking a societal perspective (including non-NHS costs) reduced the cost saving to £63 and increased the amount of uncertainty around the incremental cost, such that there was no significant difference in costs (p=0.143). Excluding intraoperative costs substantially reduced the cost savings from discarding the nail and meant that the cost difference was no longer statistically significant (p≥0.238; exploratory analysis). Changing the way that consultation costs were captured had minimal impact (exploratory analysis). Including infections that were identified after the seven-day check-up increased the difference in infections to 0.0437 per patient (95% CI: -0.0053, 0.0925; p=0.08), but had no impact on costs (exploratory analysis). However, all sensitivity analyses found the replace group to be more costly and have more infections than the discard group and found the probability of being cost-effective if society is willing to pay ≤£10,000 per infection avoided to be less than 12%.

***Results of the cost-utility analysis***

Mean NHS costs for children under 2 years were similar to those in the full sample and were £69.41 higher in the replace group than the discard group (95% CI: £13.45, £133.78; p=0.013). The cost-utility analysis therefore confirmed the base case finding that nail replacement is dominated by discard, being more costly and less effective. There was slightly more uncertainty around the results compared with the cost-effectiveness analysis on the full sample. Bootstrapping shows that there is a 70.82% probability that replace is dominated by discard, a 99.98% chance that replace is more costly and a 12% chance of replace being cost-effective if the NHS were willing to pay £20,000 per QALY gained (Figures S2-S3). Sensitivity analyses showed that the results were robust to changes in the methods used (Table S6).

***Strengths and weaknesses of the study***

Multiple imputation methods were employed to deal with the missing resource utilisation and health outcome data. The multiple imputation techniques produced more valid and robust results compared to other simplistic techniques recommended to deal with missing data. The large amount of missing data was the main limitation with around 55% of patients missing at least one item of resource use and a further 10% missing at least one EQ-5D questionnaire. It was also necessary to make additional assumptions because the recall periods for the 7-10-day questionnaire and the final follow-up questionnaire overlapped. Furthermore, it is possible that the cost of the suture is double-counted in the analysis, since consumables are included in the cost of operation time but also costed separately. In the cost-utility analysis, we also assumed that HRQoL changes linearly between baseline and seven days, and between seven days and four months; in practice, patients may return to the utility observed at four months much earlier. However, none of these assumptions are likely to have changed the conclusions of the analysis since the cost difference is driven by the three-minute difference in operation time.

Finally, the NINJA trial compared only two alternatives. Although we can be confident that discarding the nail is less costly and non-inferior to nail replacement, we have made no comparison with other choices, such as no nail bed surgery. This could be addressed in future research by conducting either a multiple-arm RCT or a two-arm RCT comparing nail repair discarding the nail with no nail bed surgery. This would facilitate indirect comparisons, network meta-analysis and economic evaluations comparing all available alternatives.

As NINJA is a paediatric trial, there are many considerations around the appropriateness of the HRQoL measures applied and there is still room to improve HRQoL measurement in children in general. In particular, a validated tariff for EQ-5D-Y is needed, as are valid instruments for younger children. In principle, we could have reduced the amount of missing data on HRQoL (especially in children aged 2-4) by mapping from PedsQL to EQ5D-Y, although the only mapping algorithm published to date is age-specific and focuses on children aged seven years and over.^14^ Research is also needed to determine how much the NHS should be willing to pay to avoid a nail bed infection.

**Supplementary Figures and Tables**

**Table S1.** Unit costs: 2019 UK £

| **Resource** | **Unit** | **Cost per unit (£)** | **Source** |
| --- | --- | --- | --- |
| **Operation** |  |  |  |
| Cost of operation time for plastic surgery & burns | Minute | 15.896 | Public Health Scotland^3^ |
| ***Sutures for nail bed repair*** | | | |
| 5/0 Vicryl Rapide™ | Each | 4.461 | Average of the 45 cm sutures with reverse cutting needles^6^ |
| 6/0 Vicryl Rapide™ | Each | 4.685 | W9913^6^ |
| 7/0 Vicryl Rapide™ | Each | 12.252 | W9914^6^ |
| 8/0 Vicryl Rapide™ | Each | 8.706 | W9916^6^ |
| 5/0 Velosorb™ | Each | 3.662 | Average of the two with reverse cutting needles^6^ |
| 6/0 Velosorb™ | Each | 4.917 | Average of the two Velosorb™ 6/0 sutures^6^ |
| 7/0 Velosorb™ | Each | 11.352 | FKV089^6^ |
| 6/0 Polysorb™ | Each | 3.41 | Average of the 45 cm sutures with reverse cutting needles^6^ |
| 6/0 PDS | Each | 4.65 | W9860h^6^ |
| 6/0 and 7/0 Velosorb™ | Each | 16.269 | Sum of 2 rows above |
| 6/0 and 7/0 Vicryl Rapide™ | Each | 16.937 | Sum of 2 rows above |
| 6/0 Velosorbfast | Each | 4.917 | Costed as Velosorb™ 6/0 |
| Repair suture not specified | Each | 4.69 | Assumed to be 6/0 Vicryl Rapide™ as this is most common |
| Cost of a figure-of-eight Vicryl Rapide™ suture used to replace the nail | Each | 4.274 | NHS Supply Chain^6^ |
| ***Medication*** |  |  |  |
| Ibuprofen | 100mg/5ml | 1.57 | NHS Electronic Drug Tariff, Jan 2020^27^ |
| Ibuprofen | 200mg/5ml | 3.49 | NHS Electronic Drug Tariff, Jan 2020^27^ |
| Amoxicillin | 250mg/5ml | 1.18 | NHS Electronic Drug Tariff, Jan 2020^27^ |
| Amoxicillin | 500mg | 5.48 | NHS Electronic Drug Tariff, Jan 2020^27^ |
| Cefalexin | 125mg/5ml | 0.84 | NHS Electronic Drug Tariff, Jan 2020^27^ |
| Cefuroxime | 125mg/5ml | 5.2 | BNF, 11 Jan 2020^8^ |
| Chloramphenicol | 1% | 1.66 | NHS Electronic Drug Tariff, Jan 2020^27^ |
| Co-Amoxiclav | 250/62mg/5ml | 5 | NHS Electronic Drug Tariff, Jan 2020^27^ |
| Erythromycin | 125mg/5ml | 5.37 | NHS Electronic Drug Tariff, Jan 2020^27^ |
| Flucloxacillin | 125mg/5ml | 18.84 | NHS Electronic Drug Tariff, Jan 2020^27^ |
| **Outpatient care** |  |  |  |
| Plastic or hospital dressing clinic | per visit | £205.00 | Curtis & Burns^7^ – page 68 |
| Radiology (x-rays) | per test | £31.72 | NHS Reference Costs 2017/18^28^: DAPF, inflated to 2018/19 values using HCHS pay and prices index^7^ |
| Emergency department (due to nail injury) | per visit | £139.14 | NHS Reference Costs 2017/18,^28^ inflated to 2018/19 values using HCHS pay and prices index^7^ |
| **Community care** |  |  |  |
| General Practitioner (surgery visit) | per visit | £39.00 | Curtis & Burns^7^ – page 120 |
| Practice nurse (surgery visit) | per consultation | £10.85 | Curtis & Burns^7^ – page 118. |
| Calls to NHS 111 (formally NHS Direct) | per call | £14.32 | The Financial Times,^9^ inflated to 2018/19 values using HCHS pay and prices index^7^ |
| Minor injuries | per attendance | £68.55 | NICE,^10^ inflated to 2018/19 values using HCHS pay and prices index^7^ |

Abbreviations: BNF, British National Formulary; HCHS, hospital & community health services; NHS, National Health Service; NICE, National Institute for Health and Clinical Excellence

**Table S2:** Multiple imputation model specification

| **Variables** | **Imputation function** | **Explanatory variables** | **Rationale** |  |
| --- | --- | --- | --- | --- |
| Age | N/A | N/A | Baseline covariates |  |
| Gender | N/A | N/A | Baseline covariates |  |
| Treatment allocation | N/A | N/A | Treatment indicator |  |
| Antibiotic cost up to 7-days | N/A | N/A | Complete data as these were captured in 3 different case report forms. Expected correlation with cost and infection. |  |
| Cost of the suture used to repair the nail bed injury | Regress, pmm | Full model | Cost component |  |
| Duration of time for which the tourniquet was applied during the operation | Regress, pmm | Full model | Strong predictor of operation time |  |
| Operation time | Regress, pmm | Full model | Data to calculate cost component |  |
| Any problem at 4-month | Logit | Full model | To condition that no health resource was use if there was no problem reported |  |
| NHS cost at 4-month: cost of healthcare consultations and painkiller descriptions | Regress, pmm | Full model, condition on any problem | Cost component |  |
| Non-NHS cost at 4-month | Regress, pmm | Full model | Cost component |  |
| NHS cost at 7-day: cost of healthcare consultations and painkiller descriptions | Regress, pmm | Full model | Cost component |  |
| Non-NHS cost at 7-day | Regress, pmm | Full model | Cost component |  |
| Antibiotic cost post 7-day | Regress, pmm | Full model | Cost component |  |
| **Imputation model for cost-effectiveness analysis^1^** | | | | |
| 7-day infection | Logit | Full model, omit any problem, repair suture cost, non-NHS4m, NHScost4m | Health outcome  Omitted the variables specified due to perfect prediction |  |
| **Imputation model for cost-utility analysis^2^** | | | | |
| Baseline utility | Single mean imputation stratified by groups | N/A | Strong predictor of later health state and independent of baseline covariates |  |
| Utility at 7-day | Regress, pmm | Full model | Health outcome |  |
| Utility at 4-month | Regress, pmm | Full model | Health outcome |  |
| 1: included all above listed variable  2: included all above listed variable except infection  pmm, predictive mean matching | | | | |

**Table S3:** Costs and resource use calculated on an available case basis (omitting patients with missing data for that resource)

|  |  | **Replace group** | | **Discard group** | |
| --- | --- | --- | --- | --- | --- |
|  |  | **N** | **Mean (standard deviation)** | **N** | **Mean (standard deviation)** |
| ***Consultation cost from baseline to 7 days from the seven-day form*** | | | | | |
| GP visits | No. | 133 | 0.0301 (0.1714) | 127 | 0.0 (0.0) |
|  | Cost | 133 | £1.17 (£6.69) | 127 | £0.0 (£0.0) |
| Practice nurse visits | No. | 133 | 0.0451 (0.2420) | 127 | 0.0079 (0.0887) |
|  | Cost | 133 | £0.49 (£2.63) | 127 | £0.09 (£0.96) |
| 111 calls | No. | 133 | 0.0376 (0.2862) | 127 | 0.0 (0.0) |
|  | Cost | 133 | £0.54 (£4.10) | 127 | £0.0 (£0.0) |
| Minor injuries clinic | No. | 133 | 0.0 (0.0) | 127 | 0.0079 (0.0887) |
|  | Cost | 133 | £0.0 (£0.0) | 127 | £0.54 (£6.08) |
| Plastics outpatient (excluding 7-day check) | No. | 133 | 0.0602 (0.3429) | 127 | 0.0157 (0.1250) |
|  | Cost | 133 | £12.33 (£70.29) | 127 | £3.23 (£25.62) |
| Radiology | No. | 133 | 0.0 (0.0) | 127 | 0.0157 (0.1250) |
|  | Cost | 133 | £0.0 (£0.0) | 127 | £0.50 (£3.96) |
| A&E | No. | 133 | 0.0226 (0.1490) | 127 | 0.0236 (0.1525) |
|  | Cost | 133 | £3.14 (£20.74) | 127 | £3.29 (£21.21) |
| Physiotherapy | No. | 133 | 0.0 (0.0) | 127 | 0.0 (0.0) |
|  | Cost | 133 | £0.0 (£0.0) | 127 | £0.0 (£0.0) |
| ***Operation cost*** | | | | | |
| Operation time | Minutes | 128 | 23.02 (11.14) | 123 | 18.42 (9.68) |
|  | Cost | 128 | £365.88 (£177.02) | 123 | £292.87 (£153.84) |
| Cost of sutures for nail bed repair | Cost | 132 | £5.51 (£2.89) | 127 | £4.94 (£2.32) |
| Cost of suture for replacing nail | Cost | 133 | £4.08 (£0.89) | 127 | £0.03 (£0.38) |
| Cost of 7-day check | Cost | 133 | £198.83 (£35.15) | 127 | £203.39 (£18.19) |
| ***Indirect health cost from baseline to 7 days from the seven-day form*** | | | | | |
| Paid work loss | Cost | 131 | £63.36 (£164.39) | 125 | £58.0 (£144.47) |
| Financial loss | Cost | 133 | £42.62 (£119.56) | 127 | £28.40 (£81.63) |
| ***Drug cost from baseline to 7 days from the seven-day form*** | | | | | |
| Antibiotics | Cost | 133 | £4.04 (£5.26) | 127 | £3.42 (£5.13) |
| NHS medication | Cost | 133 | 0.3795 (2.5144) | 127 | 0.0 (0.0) |
| Non-NHS medication | Cost | 131 | £2.64 (£6.04) | 126 | £2.38 (£5.78) |
| ***Consultation costs from baseline to 4 months from the 4 months form*** | | | | | |
| GP visits | No. | 150 | 0.040 (0.2282) | 150 | 0.0133 (0.1151) |
|  | Cost | 150 | £1.56 (£8.90) | 150 | £0.52 (£4.49) |
| Practice nurse visits | No. | 150 | 0.040 (0.2809) | 150 | 0.0133 (0.1633) |
|  | Cost | 150 | £0.43 (£3.05) | 150 | £0.14 (£1.77) |
| 111 calls | No. | 150 | 0.0 (0.0) | 150 | 0.0 (0.0) |
|  | Cost | 150 | £0.0 (£0.0) | 150 | £0.0 (£0.0) |
| Minor injury | No. | 150 | 0.0067 (0.0816) | 150 | 0.0 (0.0) |
|  | Cost | 150 | £0.46 (£5.60) | 150 | £0.0 (£0.0) |
| Plastics outpatient | No. | 150 | 0.0133 (0.1151) | 150 | 0.0333 (0.2696) |
|  | Cost | 150 | £2.73 (£23.59) | 150 | £6.83 (£55.28) |
| Pathology outpatient | No. | 150 | 0.0 (0.0) | 150 | 0.0 (0.0) |
|  | Cost | 150 | £0.0 (£0.0) | 150 | £0.0 (£0.0) |
| Radiology outpatient | No. | 150 | 0.0 (0.0) | 150 | 0.0 (0.0) |
|  | Cost | 150 | £0.0 (£0.0) | 150 | £0.0 (£0.0) |
| Physiology outpatient | No. | 150 | 0.0067 (0.0816) | 150 | 0.0 (0.0) |
|  | Cost | 150 | £0.67 (£8.16) | 150 | £0.0 (£0.0) |
| A&E | No. | 150 | 0.0267 (0.2302) | 150 | 0.0 (0.0) |
|  | Cost | 150 | £3.71 (£32.03) | 150 | £0.0 (£0.0) |
| ***Indirect health cost from baseline to 4 months from the 4 months form*** | | | | | |
| Paid wok loss | Cost | 119 | £27.31 (£125.51) | 119 | £45.29 (£179.97) |
| Financial cost | Cost | 123 | £20.93 (£125.07) | 120 | £36.45 (£215.20) |
| ***Drug cost from baseline to 4 months from the 4 months form*** | | | | | |
| Antibiotics | Cost | 151 | £0.07 (£0.47) | 149 | £0.04 (£0.38) |
| NHS medication | Cost | 152 | £0.0 (£0.0) | 153 | £0.0 (£0.0) |
| Non-NHS medication | Cost | 151 | £0.32 (£2.24) | 149 | £0.10 (£1.24) |

**Figure S1:** Scatter graph showing the base case economic evaluation results on the cost-effectiveness plane. The dotted lines represent different values for the value (R) that the NHS might be willing to pay to avoid one nail bed infection. Each green dot represents one bootstrap. The proportion of bootstraps that lie below or to the right of the diagonal lines represents the probability that replacing the nail after nail bed injury is cost-effective and is plotted on Figure 1.

**
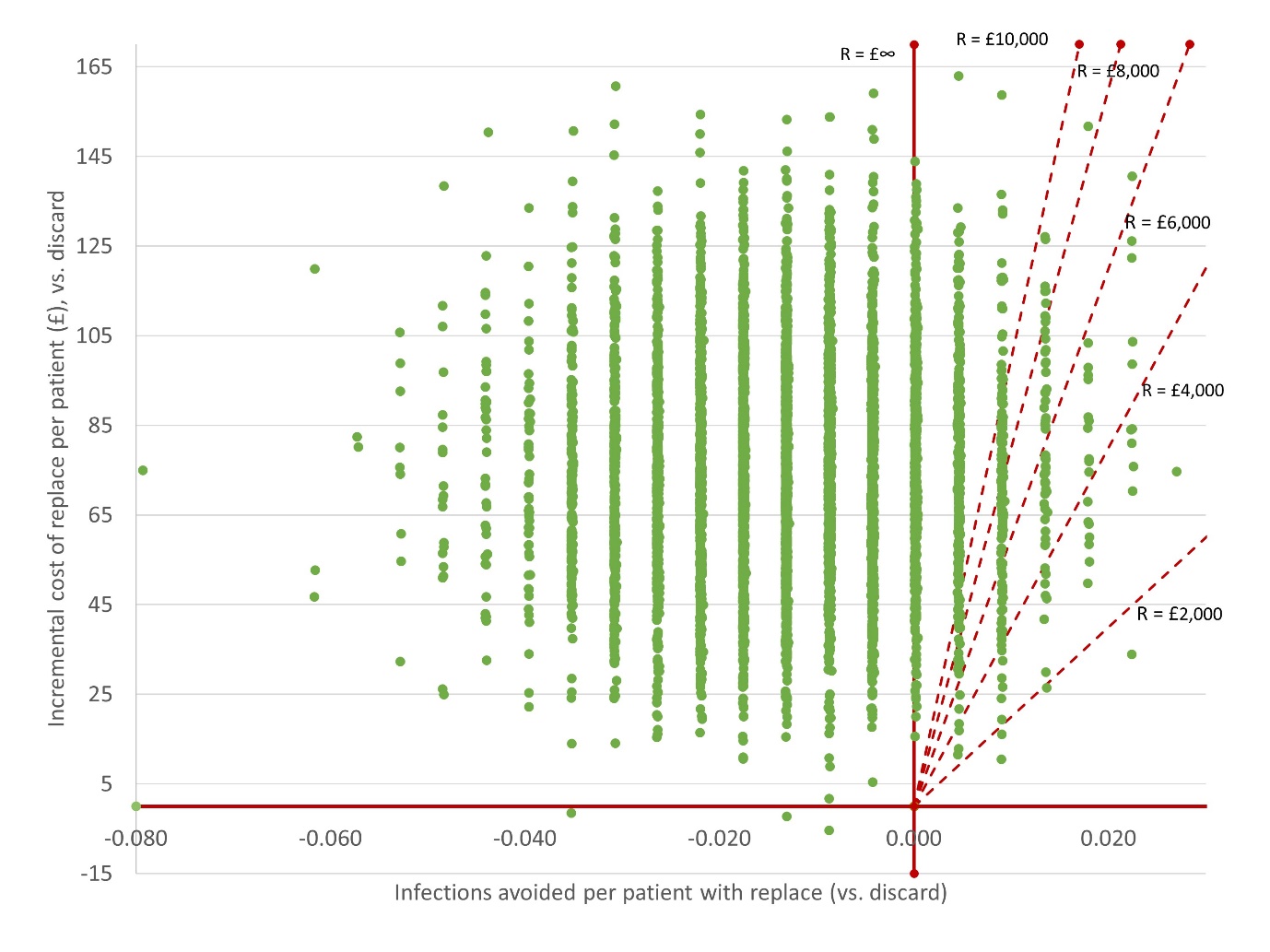
**

**Table S4:** Results of the sensitivity analyses on the cost-effectiveness analysis. All values are based on bootstrapping following multiple imputation.

|  | **Mean (95% CI)** | | | **Probability that replace is** | |
| --- | --- | --- | --- | --- | --- |
|  | **Nail replaced (n=227)** | **Nail discarded (n=224)** | **Difference**  **(replace minus discard)** | **Dominated by discard** | **More costly than discard** |
| Rate of infections by 7-10 days per patient | 0.0230 (0.0044, 0.0441)* | 0.0093 (0.0, 0.0223) | 0.0137 (-0.0091, 0.0352) | - | - |
| Total cost: base case analysis (NHS perspective) | £593.14 (£558.21, £635.69)* | £518.07 (£493.73, £543.97)* | £75.07 (£30.05, £124.11)* | 82.91% | 99.95% |
| ***Sensitivity analyses*** | | | | | |
| Total cost: societal perspective | £723.42 (£664.42, £790.85)* | £660.22 (£606.74, £723.89)* | £63.21 (-£21.81, £147.12) | 77.09% | 92.84% |
| Total cost: excluding intra-operative cost†¶ | £233.50 (£209.44, £268.25)* | £214.05 (£202.70, £228.65)* | £19.44 (-£9.53, £55.97) | 73.09% | 88.09% |
| Total cost: Excluding operation time‡ | £242.69 (£218.73, £277.45)* | £219.21 (£207.89, £233.84)* | £23.48 (-£5.59, £60.22) | 77.65% | 93.47% |
| Total cost: using consultation and drug data solely from 7-day form | £586.59 (£553.15, £626.07)* | £513.15 (£490.10, £537.75)* | £73.44 (£31.25, £119.33)* | 82.95% | 99.98% |
| Total cost: using consultation and drug data solely from 4-month form¶ | £586.26 (£551.35, £629.05)* | £510.45 (£486.65, £535.02)* | £75.81 (£32.10, £123.67)* | 82.93% | 99.96% |
| Total cost: including cost of unrelated hospital admission§¶ | £593.14 (£558.21, £635.69)* | £531.37 (£498.66, £570.31)* | £61.77 (£8.82, £115.04)* | 81.91% | 99.96% |
| Total cost CEA1¥ | £590.76 (£570.20, £648.50)* | £508.08 (£497.04, £558.72)* | £82.69 (£34.88, £131.92)* | 65.70% | 100.00% |
| Rate of infections/patient: including all infections by end of trial\|\|¶ | 0.0657 (0.0308, 0.1101)* | 0.0220 (0.0, 0.0625) | 0.0437 (-0.0053, 0.0925) | 95.50% | 99.98% |

* p<0.05

† The sensitivity analysis excluding intra-operative resource use includes the cost of all of the resources from the 7 day form and the cost of the 7 day check, but excludes sutures and operation time.

‡ Includes all costs that are in the base case analysis except operation time (matching the statistical and health economics analysis plan^1^).

§ Total cost including unrelated hospital admission sensitivity analysis: This is identical to the base case analysis, except for including the cost of one paediatric hospital admission for 1 patient in the discard group who was hospitalised for a week for an SAE related to the operation, but not related to the intervention.

¥ The cost-effectiveness analysis 1 (CEA1) specified in the statistical and health economics analysis plan^1^ comprised a complete case analysis (excluding any patients with missing data on 7-day infections or resources at 7-10 days), took a 7-10 day time horizon and excluded the cost of all drugs (including antibiotics).

|| The sensitivity analysis including infections up to the end of the trial was based on a separate run of multiple imputation. This analysis should be interpreted with caution due to the amount of missing data on infections beyond 7-10 days and the limitations of the imputation function. No patients in the discard group reported new infections after the 7-10 day follow-up.

¶ Exploratory analysis not specified in the statistical and health economic analysis plan.^1^

**Table S5:** Result for EQ-5D-Y for available case and imputed data, excluding children under 2 years old (with no adjustment for baseline utility)

|  | **Replace group** | | **Discard group** | |
| --- | --- | --- | --- | --- |
|  | **n^*^** | **Mean (Standard deviation)** | **n** | **Mean (Standard deviation)** |
| ***Available case analysis*** | | | | |
| Baseline | 139 | 0.6961 (0.2961) | 148 | 0.6907 (0.2697) |
| 7 days | 78 | 0.7641 (0.2166) | 79 | 0.7638 (0.2134) |
| 4 months | 77 | 0.9452 (0.1044) | 79 | 0.9696 (0.0755) |
| Utility change between baseline and 4 months | 77 | 0.2337(0.2642) | 79 | 0.2860 (0.2647) |
| QALYs up to 4 months | 59 | 0.2796 (0.0435) | 60 | 0.2873 (0.0405) |
| ***With multiple imputation of post-baseline values and mean imputation of baseline values*** | | | | |
| Baseline | 158 | 0.7029 (0.2776) | 165 | 0.6953 (0.2554) |
| 7 days | 158 | 0.7593 (0.2182) | 165 | 0.7671 (0.2129) |
| 4 months | 158 | 0.9528 (0.0967) | 165 | 0.9617 (0.0856) |
| Utility change between baseline and 4 months | 158 | 0.1249 (0.1374) | 165 | 0.1332 (0.1297) |
| QALYs up to 4 months | 158 | 0.2829 (0.0422) | 165 | 0.2856 (0.0404) |

QALY, quality-adjusted life-year.

**Table S6:** Results of the cost-utility analysis and sensitivity analyses. All values exclude patients aged under 2 years and are based on bootstrapping following multiple imputation.

|  | **Mean (95% CI)** | | | | **Probability that replace is** | | |
| --- | --- | --- | --- | --- | --- | --- | --- |
|  | **Nail Replaced (n=158)** | **Nail Discarded (n=165)** | **Difference**  **(replace minus discard)** | **Domin-ated by discard** | | **More costly than discard** | |
| QALYs gained by 4 months | 0.2826 (0.2733, 0.2907) * | 0.2859 (0.2777, 0.2934) * | -0.0034 (-0.0150, 0.0082) |  | | |  |
| NHS perspective (base case) | £595.86 (£549.44, £654.18) * | £526.46 (£497.85, £557.03) * | £69.41 (£13.45, £133.78) * | 70.82% | | | 99.98% |
| Total cost: societal perspective | £734.71 (£663.10, £823.80) * | £665.75 (£601.93, £744.03) * | £68.96 (-£35.88, £176.49) | 64.49% | | | 99.53% |
| Total cost: excluding intra-operative cost†¶ | £237.80 (£204.71, £289.27) * | £215.64 (£201.21, £233.85) * | £22.16 (-£17.51, £75.94) | 59.26% | | | 89.02% |
| Total cost: Excluding operation time‡ | £246.83 (£213.72, £298.41) * | £220.69 (£206.23, £239.02) * | £26.15 (-£13.45, £80.01) | 63.09% | | | 94.75% |
| Total cost: using consultation and drug data solely from 7-day form | £584.21 (£542.41, £635.58) * | £517.67 (£491.48, £545.96) * | £66.53 (£15.57, £122.88) * | 70.91% | | | 99.98% |
| Total cost: using consultation and drug data solely from 4-month form¶ | £595.11 (£548.25, £653.17) * | £520.70 (£492.46, £551.87) * | £74.41 (£17.27, £138.34) * | 71.02% | | | 99.98% |
| Total cost: including cost of unrelated hospital admission§¶ | £595.86 (£549.44, £654.18) * | £544.50 (£503.85, £596.78) * | £51.36 (-£18.08, £123.52) | 66.03% | | | 99.98% |

* p<0.05

† The sensitivity analysis excluding intra-operative resource use includes the cost of all of the resources from the 7 day form and the cost of the 7 day check, but excludes sutures and operation time.

‡ Includes all costs that are in the base case analysis except operation time (matching the statistical and health economics analysis plan^1^).

§ Total cost including unrelated hospital admission sensitivity analysis: This is identical to the base case analysis, except for including the cost of one paediatric hospital admission for 1 patient in the discard group who was hospitalised for a week for an SAE related to the operation, but not related to the intervention.

¶ Exploratory analysis not specified in the statistical and health economic analysis plan.^1^

**Figure S1:** Scatter graph showing the results of the cost-utility analysis on the cost-effectiveness plane. The dotted lines represent different values for the value that the NHS might be willing to pay to avoid one nail bed infection. Each green dot represents one bootstrap. The proportion of bootstraps that lie below or to the right of the diagonal lines represents the probability that replacing the nail after nail bed injury is cost-effective and is plotted on Figure S3.

**Figure S2:** Cost-effectiveness acceptability curves for the cost-utility analysis and sensitivity analyses. In order to highlight differences between analyses, the y-axis shows only the 0-0.3 range.

**References for supplemental materials**

1. Stokes JR, Png ME, Jain A, Greig AVH, Shirkey BA, Dritsaki M, et al. Should the nail plate be replaced or discarded after nail bed repair in children? Nail bed INJury Analysis (NINJA) randomised controlled trial: a health economic and statistical analysis plan. Trials. 2020;21(1):833.

2. Greig A, Gardiner MD, Sierakowski A, Zweifel CJ, Pinder RM, Furniss D, et al. Randomized feasibility trial of replacing or discarding the nail plate after nail-bed repair in children. British Journal of Surgery. 2017;104:1634-9.

3. Public Health Scotland. Theatres: Costs - Detailed Tables. R142X - average theatre running costs, and usage by specialty, by board 2019 [Available from: <https://www.isdscotland.org/Health-Topics/Finance/Costs/Detailed-Tables/Theatres.asp> Accessed 25 Nov 2020].

4. Jain A, Greig A, Jones A, Cooper C, Davies L, Greshon A, et al. Effectiveness of nail bed repair in children with or without replacing the fingernail: the NINJA multicentre randomised controlled trial. British Journal of Surgery. 2023;(in press).

5. National Health Service. Reference cost 2017/18 [Available from: <https://improvement.nhs.uk/resources/reference-costs/> Accessed 25 Nov 2020].

6. National Health Service. NHS supply chain catalogue 2020 [Available from: <https://my.supplychain.nhs.uk/catalogue> Accessed 25 Nov 2020].

7. Curtis LA, Burns A. Unit Costs of Health and Social Care 2019, PSSRU, Kent, UK, 176 pp 2019 [Available from: <https://www.pssru.ac.uk/project-pages/unit-costs/unit-costs-2019/> Accessed

8. Joint Formulary Committee. BNF for Children: London: Pharmaceutical Press.; 2020 [11 Jan 2020:[Available from: <http://www.medicinescomplete.com> Accessed 25 Nov 2020].

9. Financial Times. NHS to trial artificial intelligence app in place of 111 helpline 2017 [Available from: <https://www.ft.com/content/aefee3b8-d1d8-11e6-b06b-680c49b4b4c0> Accessed 25 Nov 2020].

10. NICE. Emergency and acute medical care in over 16s: service delivery and organisation, Chapter 18 2018 [Available from: <https://www.nice.org.uk/guidance/ng94/evidence/18.minor-injury-unit-urgent-care-centre-or-walkin-centre-pdf-172397464605> Accessed 25 Nov 2020].

11. Wille N, Badia X, Bonsel G, Burström K, Cavrini G, Devlin N, et al. Development of the EQ-5D-Y: a child-friendly version of the EQ-5D. Quality of life research. 2010;19(6):875-86.

12. Alderfer MA, Marsac ML. Pediatric Quality of Life Inventory (PedsQL). 2013.

13. Varni JW, Limbers CA, Burwinkle TM. How young can children reliably and validly self-report their health-related quality of life?: An analysis of 8,591 children across age subgroups with the PedsQL™ 4.0 Generic Core Scales. Health and quality of life outcomes. 2007;5(1):1-.

14. Khan KA, Petrou S, Rivero-Arias O, Walters SJ, Boyle SE. Mapping EQ-5D utility scores from the PedsQL™ generic core scales. Pharmacoeconomics. 2014;32(7):693-706.

15. Chen G, Ratcliffe J. A Review of the Development and Application of Generic Multi-Attribute Utility Instruments for Paediatric Populations. PharmacoEconomics. 2015;33(10):1013-28.

16. Dolan P, Gudex C, Kind P, Williams A. Valuing health states: A comparison of methods. Journal of health economics. 1996;15(2):209-31.

17. Manca A, Hawkins N, Sculpher MJ. Estimating mean QALYs in trial-based cost-effectiveness analysis: the importance of controlling for baseline utility. Health economics. 2005;14(5):487-96.

18. Faria R, Gomes M, Epstein D, White I. A Guide to Handling Missing Data in Cost-Effectiveness Analysis Conducted Within Randomised Controlled Trials. 2014;32(12):1157-70.

19. Schomaker M, Heumann C. Bootstrap inference when using multiple imputation. Statistics in medicine. 2018;37(14):2252-66.

20. Dakin HA, Leal J, Briggs A, Clarke P, Holman RR, Gray A. Accurately Reflecting Uncertainty When Using Patient-Level Simulation Models to Extrapolate Clinical Trial Data. Medical decision making. 2020;40(4):0272989X2091644-473.

21. White IR, Royston P, Wood AM. Multiple imputation using chained equations: Issues and guidance for practice. Statistics in medicine. 2011;30(4):377-99.

22. Bono R, Blanca MJ, Arnau J, Gómez-Benito J. Non-normal Distributions Commonly Used in Health, Education, and Social Sciences: A Systematic Review. Frontiers in psychology. 2017;8:1602.

23. Schlittgen R. Analysis of incomplete multivariate data. Computational statistics & data analysis. 1999;30(4):478-9.

24. Raphael K. Recall Bias: A Proposal for Assessment and Control. International journal of epidemiology. 1987;16(2):167-70.

25. Schmier JK, Halpern MT. Patient recall and recall bias of health state and health status. Expert Review of Pharmacoeconomics & Outcomes Research. 2004;4(2):159-63.

26. Rubin DB, Toutenburg, H. Multiple imputation for nonresponse in surveys: Wiley, New York 1987. XXIX+258 pp. Statistical papers (Berlin, Germany). 1990;31(1):180-.

27. National Health Service. Electronic Drug Tariff 2020 [Available from: <http://www.drugtariff.nhsbsa.nhs.uk/#/00770298-DC/DC00770293/Home> Accessed 25 Nov 2020].

28. National Health Service. Reference cost 2017/18 2019 [Available from: <https://improvement.nhs.uk/resources/reference-costs/> Accessed 25 Nov 2020].
